# Emotional meanings of clinical practice with head and neck cancer patients: a qualitative study with Brazilian professionals

**DOI:** 10.1016/j.bjorl.2026.101923

**Published:** 2026-10-14

**Authors:** Adriana Consuelo Oliveira Bispo, Rodrigo Almeida Bastos, Ricardo Souza Evangelista Sant'Ana, Sarah Adriely da Silva, Milena Rossi Suedt, Jéssica Renata Ponce-de-Leon, Claudiane Graças dos Santos, Luciane Miranda Guerra, Carmen Silvia Passos Lima, Egberto Ribeiro Turato

**Affiliations:** a Laboratory of Clinical-Qualitative Research, School of Medical Sciences, University of Campinas, Campinas, São Paulo, Brazil; b School of Nursing of Ribeirão Preto, University of São Paulo, Ribeirão Preto, São Paulo, Brazil; c School of Odontology, University of Campinas, Piracicaba, São Paulo, Brazil; d Clinical Oncology Service, Department of Anesthesiology, Oncology, and Radiology, School of Medical Sciences, University of Campinas, Campinas, São Paulo, Brazil

**Keywords:** Head and neck cancer, Healthcare personnel, Qualitative research, Lived experiences, Clinical practice

## Abstract

**Objective:**

To interpret emotional meanings attributed by healthcare professionals to the clinical management of patients with head and neck cancer, in a university-specialised outpatient service at a public general hospital.

**Method:**

A qualitative study was carried out using semi-directed interviews with open-ended questions in-depth. The sample was closed by the saturation strategy. Data treatment was conducted by Clinical-Qualitative Content Analysis technique under theoretical framework of psychodynamic concepts from Medical Psychology, and validation by peers from a research group.

**Results:**

Ten clinical professionals were interviewed: six nurses and four physicians. From the analysis of the interviews, three categories are constructed for this article: (1) An instituted stigmatization; (2) Wide-open reality; (3) Naturalization without trivialization. The aggressiveness of these tumours, the association with harmful lifestyle habits, and the great concern of family members call for clinical-psychological management to mitigate emotional distress, to handle possible guilt, and prevent any burnout, improving a better adherence to treatment and harmonious interpersonal relationships.

**Conclusion:**

The stigma is a symbolic entity that takes distress for the clinicians but also seems to highlight a higher level of awareness and reflection. This awareness can be a starting point for the development of healthier strategies for emotional coping, contributing to a more balanced work and personal harmonic relationship of care.

**Level of evidence:**

5.

## Highlights

• Symbolic meanings related to patients contribute to health professionals’ distress.
• The perception that a patient is difficult often comes from countertransference.
• Healthcare professionals have a visual understanding of the “shape” of patients.
• Group meetings with physicians to speak on the handling of patients is efficient.

## Introduction

The relationship between clinical team and patients is dynamically established in objective and subjective contexts, where social, individual, and interpersonal aspects interface with scientific, political, and cultural factors.^1^ Two key elements in this context are the phenomena of transference and countertransference.^2^ Transference refers to the emotions and attitudes of the patient concerning the healthcare professional, often reproducing patterns from previous relationships. Countertransference refers to the emotions and reactions that healthcare professionals may experience in response to patients, often unaware. It may happen due to the characteristics of the disease, and the individual particularities of the patient or the family.^2^^,^^3^ Therefore, transference originates in the patient, while countertransference is recognized and managed by the healthcare professional. However, these emotions are not always managed by healthcare professionals, exposing them to distress when they experience situations that go beyond the disease, challenging them in the context of the healthcare professional-patient relationship.^3^

Health professionals experience an emotional burden with several patients, each with their unique way of handling the disease.^4^ In the case of professionals who treat patients with Head and Neck Cancer (HNC), these are recurring experiences.

HNC predominantly affects male individuals over 50-years of age with a history of high tobacco and alcohol consumption.^5^^,^^6^ This context follows international statistics, which list HNC as the sixth most common cancer worldwide, with 878,348 new cases and 444,347 deaths estimated in 2020.^7^^,^^8^ The treatment is complex and involves several specialists in a therapeutic proposal to minimize the impacts of interventions and improve the quality of life.^5^^,^^9^

The analysis of these complex emotional processes of healthcare professionals in the relationship with HNC patients can provide important insights into the underlying perceptions and attitudes of these patients.^10^^,^^11^ It allows healthcare professionals to improve their understanding of the patient’s individual needs and emotional reactions to diagnosis and treatment. This in-depth understanding can support the formulation of personalized therapeutic strategies, contributing to more appropriate and effective clinical decisions.^11^

Studies show that investing in support for the healthcare team can enrich the quality of therapeutic relationships, promoting a more empathetic and collaborative environment between healthcare professionals and patients.^12^^,^^13^ By recognizing and appropriately managing the personal emotions involved in the relationship with the patient, healthcare professionals and institutions can promote a more compassionate and sensitive approach to care, resulting in a more humanized experience for the patient during the treatment process.^12^ In this context, to suppress distress resulting from the HNC patient’s situation of fragility and vulnerability, health professionals end up facing some emotional disorganization.^4^ By not understanding this phenomenon, the healthcare teams feel emotional exhaustion, which may influence clinical decisions and the process of building therapeutic relationships for humanized care.^4^, ^5^, ^6^

This study aimed to interpret the perceptions reported and meanings attributed by health professionals to the clinical management of patients with HNC in a specialised clinical service at a general hospital of a Brazilian public university.

## Methods

### Patients

This is a humanistic study using the Clinical-Qualitative Method (CQM) described by Turato,^14^ which interprets psychodynamically the reports of study subjects about their experiences in clinical settings. The study was approved by the Research Ethics Committee of the University of Campinas and Brazil’s National Health Council under nº 09048919.7.0000.5404. Healthcare professionals who agreed to participate in this study signed an informed consent form, within an empathetic relationship in a private room.

Sample selection was intentional and sequential, using the information saturation criterion described by Fontanella and collaborators^15^ to close the group of persons from the staff. The inclusion criteria were: (1) Nurse in the clinical oncology outpatient service for more than 6-months; (2) Resident physician in the third year of residency at the University Hospital; and (3) Provide care to HNC patients in all wards of the clinical oncology outpatient service of the university hospital. There was no sample loss.

Data were collected using Semi-Directed Interviews with Open-ended Questions in-Depth ‒ SDIOQD, in the clinical oncology service. SDIOQD is a conversation, an eye-to-eye Interview. It is Semi-Directed because the researcher provides initial direction with a trigger question. From then on, the interviewee takes the lead. The questions are Open-Ended, as the answers follow the interviewee's free association of ideas. It is in-Depth because the interviewee is encouraged to expand on their report. The interviews were conducted by the first author of this article, a female nurse. The speeches were audio-recorded and transcribed in full by a scholarship student. Additional observations, perceptions, and reactions of the participants and the researcher during the interviews were written in field notes, such as paraverbal and nonverbal languages, which were used when required to complement relevant information. The average duration of the ten interviews was 35 minutes.

Data processing was conducted according to the Seven Steps of Clinical-Qualitative Content Analysis by Faria-Schützer and collaborators^16^: (1) Editing of the material for analysis – transcription of whole interviews made by the researcher; (2) Free-floating reading – search for cores of sense; (3) Construction of units of analysis – identification of meanings, selection of speech excerpts, reflections on each fragment; (4) Construction of meaning codes – a grouping of similar units of analysis to structure the first meaning codes; (5) Construction of categories – organization of materials for analysis of all interviewees to group the meaning codes; (6) Discussion – a dialogue with literature; and (7) Validity – critical reflection on the processes conducted in each stage – it is performed by all authors using the Standards for Reporting Qualitative Research (SRQR).^10^ The theoretical framework used as support was the Balintian British Medical Psychology, which explores psychodynamic concepts of the professional-patient emotional relationship.^2^ The final validation occurred in meetings of the Lab. of Clinical-Qualitative Research (LCQR) with peers who also conduct investigations with the CQM. The research group, which has been in existence for almost three decades, is composed of two senior investigators and presently master's and doctoral students who meet weekly.

## Results

### Characteristics of study participants

Ten professionals were interviewed, six nurses and four physicians, with a minimum and a maximum professional experience of 5-years and 32-years, respectively, and a minimum and a maximum professional experience with HNC patients of 1-year, 14-years, respectively (Table 1).

**Table 1:** Biodemographic characteristics of the participants, Campinas SP, Brazil, 2021.

Number	Sex/ Gender	Occupation	Experience in months	Experience with HNC patients in months
P1	F	Nurse	16	14
P2	F	Nurse	32	4
P3	F	Nurse	9	7
P4	F	Nurse	7	3
P5	F	Nurse	30	3
P6	F	Nurse	12	1
P7	M	Physician	8	3
P8	F	Physician	5	3
P9	F	Physician	6	3
P10	F	Physician	6	3

### Data categorization

From the analysis of the interviews, three categories were constructed for this paper: (1) An instituted stigmatization; (2) Wide-open reality; and (3) Naturalization without trivialization.

### An instituted stigmatization

This category is based on the perspective of healthcare professionals regarding the behavioural factors that contribute to the patient’s disease. It covers observations and perceptions about the disease stage and the social interactions involved. Also, healthcare professionals identify and emphasize the situations they will have to handle, revealing the subjective elements and the challenges involved in the relationship with a 'difficult patient’.

The self-destructive lifestyle habits subcategory is based on the perspective of healthcare professionals regarding behavioural factors.

“It is the classic profile of men, cigarettes and alcohol involved and sometimes drugs. […] Another thing we realize is that the patient is marginalized.” (P1)

“He is a very stigmatized patient. I think he is stigmatized because of his lifestyle when compared to other tumours […]. A head and neck tumour sometimes doesn’t have that empathy and can even involve some contempt, let’s say.” (P8)

The patient’s epidemiological profile is analysed, together with the social implications of this profile and how such implications affect the dynamics of the relationship between the patient and healthcare professionals. Healthcare professionals act as observers, with the perception that patients may be subject to stigmatization and marginalization. However, this position seems to be an attempt to avoid labelling the patient, which generates emotional tension due to the conflict between this perception and the desire to adopt a less stigmatizing approach.

This narrative is influenced by ethical considerations, with healthcare professionals seeking a neutral position when faced with moral judgments about the patient. However, it is important to recognize that these professionals are products of society and, therefore, are susceptible to influences from existing conceptions that shape their perception of patients. Given that underlying behaviours are often linked with addictions, they tend to see the patient in a one-dimensional way based on these habits. Healthcare professionals feel distressed when trying to avoid this marginalization.

The damaged family relationship subcategory reflects the feelings of healthcare professionals about the presence of fragile affective-family dynamics when interacting with patients and the lack of support from the patient’s immediate family members. This perception conflicts with the traditional concept of family as an essential support during the disease process, especially for patients with cancer.

“[…] Usually disconnected from the family, they have a neighbour, an ex-wife, a son who, let’s say, supports the patient. They go there and help the patient. Then they disappear! Most of them, not all.” (P8)

“Most of the time they are not accompanied by wives; they are cousins, uncles, friends, brothers-in-law, brothers.” (P7)

“We see the caregiver is not even from the family, it’s a neighbour, a church friend, a friend from the bar.” (P1)

The development of social dynamics associated with deep-rooted stigma is observed in clinical environments, that is, in microscopic contexts of practice. In these circumstances, there is an evident perception that the patient has irrevocably compromised family relationships. Healthcare professionals readily articulate a narrative of whether factual or symbolic nature that exposes the supposed transgression of moral values by patients with HNC, thus justifying the deterioration of family connections and fragile affectional bonds.

“Head and neck cancer patients present a certain pattern. They are often patients who smoked a lot, drank a lot, and maybe because of that, they don’t have a strong family bond, then sometimes they come alone even with difficulties.” (P7)

“The family tends to deny this patient due to the type of care, the dressing. Suddenly he was a guy who drank, he had a history of beating his wife, not giving money, spending the night drinking and using drugs. And there are families who say: ‘You see? Now he’s paying me back!’ And then the only thing you can do is listen to what they say and try to mediate it. And how can we heal this wound from the past? It’s not easy, it’s very difficult.” (P2)

While recognizing the weakened family relationships in patients with HNC, healthcare professionals also realize they cannot fully count on family support. This scenario does not enable to creation of the vital triad of healthcare professionals, patients, and families, in which family support is recognized as crucial for both the patient and healthcare professionals during the treatment process. This undesirable situation becomes more relevant when considering the patient’s family issues are added to the disease demands that need to be managed. This dynamic also affects the relationship between the healthcare professionals and the patient, as these professionals will find themselves overwhelmed, handling complex subjective issues that involve their own family values.

The shameful disease subcategory reflects the stigmatization of patients with HNC in a particular dimension in the clinical context. The term “stigmatized” is used by healthcare professionals to support the narrative of the patient’s perception of the disease, including its aesthetic issues. This term is also used to express the embarrassment shared by healthcare professionals.

“He is strongly stigmatized. The patient feels embarrassed when he is around other people, because of the odour. Strong odour and a visual issue.” (P5)

“The first impression is when you press to see the next patient in a queue, you often expect a patient with a physical deformity, sometimes with a strong odour, with devices such as a tracheal tube, a probe, wiping off drool with a towel […]. And we’ve already seen patients who came in with larvae, with myiasis.” (P7)

“I think that everyone who’s going to treat a head and neck cancer patient is nervous about how they look. I think it makes a big impression, right?” (P3)

This phenomenon is clearly illustrated in the statements of interviewees P7 and P3. Although they have understandings of the characteristics of the disease and the patient’s profile, and can handle these aspects in their routine, they still experience a feeling of surprise and apprehension regarding what they are about to see. This feeling reflects an intrinsic distress of seeing the “grotesque.” The impressive presence of characteristics such as intense odour, visible injuries, and deformations of the patient is perceived by the senses without requiring cognitive efforts.

### Wide-open reality

Providing care to patients with HNC places healthcare professionals in contact with the reality of cancer since this disease is visually impactful. The speeches of interviewees P7 and P1 indicate they prefer to treat patients with other milder types of cancer, particularly concerning the underlying aspects. In contrast, the patient’s coping with HNC is described as intricate and distressing.

“These are patients who, I think, are the prototype of cancer, when you say it, cancer! In the case of breast cancer, there are bald women with mastectomies, and head and neck cancer patients are deformed guys, very ugly things, with face tumours.” (P7)

“Sometimes it’s easier to treat patients with stomach cancer because most of the time they have a gastrostomy, or not, and they’re intact on the outside. But not the head and neck cancer patient, he often arrives with an apparent injury, with a probe, he arrives with a tracheal tube, he arrives with some external injury.” (P1)

### Naturalization without trivialization

The speeches suggest that healthcare professionals use strategies and natural attitudes to address what they regularly find, but such exposure still has mobilized them. These professionals can resort to the distancing technique, denying what they see as a mode to protect themselves against certain emotional tension.

“I kind of transcended that, I receive this patient and see beyond the injury, I worry a lot about his prognosis, but I’m not very impressed. I don’t take that with me. It’s his thing. It’s his! […]. When you see beyond the injury, then comes the person, right? Then I treat him as a person who doesn’t have an injury and that will work out. […] I talk as if the person doesn’t have that injury.” (P5)

“And I try to prepare myself to show the patient that it doesn’t matter to me whether he has a tracheal tube, whether he uses a probe, whether he smells bad or good. I try to prepare myself to treat him in the most receptive way, right? I try to prepare myself and be calm.” (P7)

“I try” is present in the speeches of all interviewees, particularly when they say how they handle aspects of the disease that cause aversion, perplexity, and psychological discomfort. It requires considerable effort and mental energy to maintain constant control over their reactions. In this context, health professionals adopt a “dehumanization” approach, repressing their feelings and instinctive reactions to maintain a humanized stance both in terms of mental processes of perception and behaviours adopted when interacting with patients with HNC.

## Discussion

It is largely known that healthcare professionals who treat cancer patients face challenges due to the complexity of the disease.^17^ These challenges may vary with the type of cancer, the influence of the patient’s social conception, and perception of the disease in the clinical context.^2^ An important aspect highlighted in this study is the stigma associated with patients with HNC. This phenomenon is observed in the speeches of interviewees when discussing the particularities of these patients, constituting a central issue. Stigma was a social construct, shaped by beliefs and perceptions that are influenced by the culture context. It refers to a negative or derogatory attribute that differentiates and diminishes the subject, which constitutes a disadvantage to this subject.^18^

Concretization of the symbolic meanings related to the patient’s status contributes to the distress of healthcare professionals, who strive to dissociate their patient perceptions from existing social conceptions. However, this relationship ends up generating a system of meanings arising from the social interaction itself.^18^^,^^19^ The narratives, which can be critical or moralistic, can be influenced by individual beliefs and unconscious fantasies, leading to a punitive and judgmental attitude towards the patient. Such reactions would occur unconsciously as a way to help healthcare professionals handle internal conflicts and personal values.^12^^,^^20^

The identification mechanism can occur more strongly with the patient’s family than with the patient himself, despite his condition of being affected by the disease. This process of non-perceived identification causes the person to project elements of their psychic structure onto another person, seeing this person as someone who can receive affection.^20^

This dynamic does not favour an authentic relationship and effective communication, resulting in damaged bonds with the patient. It can also cause a feeling of indignation and powerlessness in health professionals, triggering defense mechanisms in response to interpersonal and subjective issues that generate distress.^2^ The perception that a patient is “difficult”, and the relationship is challenging often comes from countertransference, a phenomenon that is inherent to healthcare environments, particularly in oncology clinics, which present a high emotional burden.^17^

When the negative aspects are aggravated by compromised self-care, they contribute to a representation of the “grotesque”, making healthcare professionals alarmed. This is not limited to appearance and includes the harmful effects of the disease on the human condition, resulting in distress.^2^^,^^18^ When providing care to patients with HNC, healthcare professionals have a direct visual understanding of the “shape” of the disease. These reactions can influence the healthcare professional-patient relationship due to the transference and countertransference that occur in these environments.^2^

Although it can be assumed that prior experience and knowledge about patients, this disease is so intense that professionals assume a “neutral position” trying to suppress their own emotions. The suppression involves psychic defence mechanisms that require mental energy. Considering a patient with HNC as a “difficult” patient can be a defense strategy because naming is a way of framing.^1^^,^^2^

## Conclusions

The issue of sociological stigmatization is present within micro-settings with its psychic symbolization. The perception of the “grotesque” is associated with distress among professionals but also demonstrates a higher awareness and reflection. This awareness can develop healthier emotional coping strategies, which can contribute to a more balanced work setting and promote care harmonization. This research offers theoretical material for reading by professionals, to name and interpret emerging tensions in the clinician-patient relationship. At least three theoretical implications of this new knowledge: (a) In teaching of the health professions to be included in theoretical programs of disciplines and books for undergraduate students; (b) Continuing education for professionals, such as courses with updating concepts on symbolic aspects of the illness experience and caregiving; (c) Offering concepts to enrich scientific guidance texts in global clinical care and policy of health treatment protocols. At least three practical applications of our findings: (a) Use for the psychological handling of a patient on his/her reported disease experiences by the clinical professional in conversation during a consultation setting; (b) Academic content in regular clinical discussion meetings of team professionals when interpretable subjective aspects emerge in a case in presentation; (c) Specific meetings to analyse emotional aspects in patient-professional challenging relationships, such as in the well-established Balint groups ‒ small groups of clinicians (physicians, nurses and so on) under any trained leader like the authors of qualitative studies.

## ORCID ID

Adriana Consuelo Oliveira Bispo: 0000-0001-7444-1851

Rodrigo Almeida Bastos: 0000-0002-6159-8048

Ricardo Souza Evangelista Sant'Ana: 0000-0003-3762-4362

Sarah Adriely da Silva: 0000-0001-5306-2617

Milena Rossi Suedt: 0000-0003-0359-0520

Jéssica Renata Ponce-de-Leon: 0000-0002-4787-6697

Claudiane Graças dos Santos: 0000-0003-0867-6075

Luciane Miranda Guerra: 0000-0002-7542-7717

Carmen Silvia Passos Lima: 0000-0002-1314-2345

Egberto Ribeiro Turato: 0000-0002-7857-1482

## Authors' contributions

Conceptualization, A.C.O.B. and E.R.T.; Methodology, R.A.B. and E.R.T.; Validation, C.S.P.L., S.A.S., M.R.S., J.R.P.L., C.G.S. and L.M.G.; Formal analysis, R.A.B. and R.S.E.S.; Investigation, A.C.O.B.; Resources, E.R.T.; Writing-original draft preparation, R.A.B and R.S.E.S.; Writing-review and editing, R.A.B., A.C.O.B. and R.S.E.S.; Visualization, C.S.P.L.; Supervision, E.R.T. and C.S.P.L; Project administration, E.R.T. All authors have read and agreed to the published version of the manuscript.

## Funding

We are grateful to Coordination for the Improvement of Higher Education Personnel (CAPES) and National Council for Scientific and Technological Development, CNPq, Research Productivity, [Grant # 302922/2025-3 (Lima CSP)] for financial support.

## Conflicts of interest

The authors declare no have conflicts of interest.

## Data availability statement

The datasets generated during and/or analyzed during the current study are presented in the current manuscript and are available from the corresponding author upon reasonable request.

## Institutional review board statement

The study was conducted in accordance with the Declaration of Helsinki and approved by the Research Ethics Committee of the University of Campinas (protocol nº 3.246.642 on April 05, 2019).

## Informed consent statement

Informed consent was obtained from subjects involved in the study.

## Justification for the number of authors

The number of authors exceeds the journal’s limit of seven; however, we request the inclusion of all ten authors due to their substantial and distinct contributions to the study. This work was developed by a multidisciplinary team that included oncologists, nurses, psychologists, and psychiatrists, whose expertise was essential for patient selection, data acquisition, and interpretation.

HNC, Head and Neck Cancer.
